# A Rapid and Simple Method for DNA Engineering Using Cycled Ligation Assembly

**DOI:** 10.1371/journal.pone.0107329

**Published:** 2014-09-16

**Authors:** Theodore L. Roth, Ljiljana Milenkovic, Matthew P. Scott

**Affiliations:** Departments of Developmental Biology, Genetics, Bioengineering, & Biology, Stanford University School of Medicine, Stanford, California, United States of America; Imperial College London, United Kingdom

## Abstract

DNA assembly techniques have developed rapidly, enabling efficient construction of complex constructs that would be prohibitively difficult using traditional restriction-digest based methods. Most of the recent methods for assembling multiple DNA fragments in vitro suffer from high costs, complex set-ups, and diminishing efficiency when used for more than a few DNA segments. Here we present a cycled ligation-based DNA assembly protocol that is simple, cheap, efficient, and powerful. The method employs a thermostable ligase and short Scaffold Oligonucleotide Connectors (SOCs) that are homologous to the ends and beginnings of two adjacent DNA sequences. These SOCs direct an exponential increase in the amount of correctly assembled product during a reaction that cycles between denaturing and annealing/ligating temperatures. Products of early cycles serve as templates for later cycles, allowing the assembly of many sequences in a single reaction. To demonstrate the method’s utility, we directed the assembly of twelve inserts, in one reaction, into a transformable plasmid. All the joints were precise, and assembly was scarless in the sense that no nucleotides were added or missing at junctions. Simple, efficient, and low-cost cycled ligation assemblies will facilitate wider use of complex genetic constructs in biomedical research.

## Introduction

Advances in techniques to manipulate DNA ignited biology’s genetic revolution and have underpinned modern advances in biomedical science [1]. The development of the Polymerase Chain Reaction (PCR), based on the in vitro application of thermostable DNA polymerases, enabled the amplification of any DNA sequence quickly and efficiently [2]. Techniques to assemble the amplified DNA sequences into circular plasmids for downstream applications have for many years been mostly limited to restriction digest-based assemblies [3]. Restriction endonucleases are DNA-cleaving enzymes that bind and create double-stranded (ds) DNA breaks at specific palindromic sequences, enabling cut DNA fragments to be joined using a DNA ligase into vectors digested with the same endonuclease [3]. However, restriction digestion is severely limiting. Only one or a few inserts can be introduced in controlled order into a vector. Multiple rounds of digestion, ligation, transformation, isolation, and sequencing are required for even the most routine multi-insert constructs. Ligated restriction fragments often have “scar” sequences, unwanted nucleotides left behind at joining sites, limiting user control over the final sequence. This is especially problematical, for example, when a protein-coding sequence must be preserved.

Recent advances in synthetic biology have provided more powerful and efficient in vitro genetic assembly strategies than restriction digestions [4]. Notably, methods such as Golden Gate Assembly [5], based on the ability of Type II restriction endonucleases to cleave DNA a short distance from their recognition sites, have enabled directed and oriented assembly of 4–6 DNA fragments. Golden Gate and related assembly techniques have shown great utility in high-throughput applications [6] and in hierarchical multi-step assembly strategies of highly homologous sequences such as TALE monomers [7]. A number of issues prevent more widespread adoption of Golden Gate for general cloning needs. Complex preparatory work is required to place the necessary restriction sites, the practical number of inserts is limited to about six, and the sequences designed for one assembly reaction cannot be reused for a different assembly without re-engineering restriction site placement [1].

Another recent method, Gibson (or Isothermal) Assembly [8], overcomes some of these limitations and is sequence-independent. A combination of an exonuclease, a ligase, and a polymerase replaces the need for restriction enzymes, instead allowing directed assembly of DNA fragments by introducing sequence homology between the beginnings and ends of adjacent inserts [8]. Gibson Assemblies were designed for megabase-sized DNA sequences with hundreds of base pairs of homology, but have been adapted to the kilobase and smaller scale of common laboratory applications such as protein engineering [9]. Gibson Assembly has limitations too, such as increasing inefficiency when the number of inserts increases, inability to assembly small (<100 bp) sequences, and complex and error-prone addition of homologous sequences that are needed to direct the orientation and order of the assembled product.

Thermostable polymerases were the key to PCR, and similarly thermostable ligases [10] have enabled applications based on a cycled-ligation reaction (CLR) [11]. Cycled ligations have been used for a variety of purposes [12]–[15], such as synthesizing genes using synthetic oligonucleotides [16], to directing blunt-end ligation reactions using linker oligonucleotides [17]. Notably, the high specificity of CLRs enables the detection of SNPs using a Ligase Chain Reaction (LCR) [18]. Here, we present a DNA fragment assembly method based on a LCR. We use short 40 bp Scaffold Oligonucleotide Connectors (SOCs) that enable directed, scarless, in vitro assembly of multiple DNA fragments into a transformable plasmid in a single reaction. SOCs can be re-used in alternative assembly designs. We apply these cycled ligation assemblies to construct DNA products containing many, variably sized, inserts. These cycled ligation assemblies are efficient and easy to design and run.

## Results

### Scaffold Oligonucleotide Connectors (SOCs) for Cycled Ligation Assembly (CLA)

**Figure 1:**
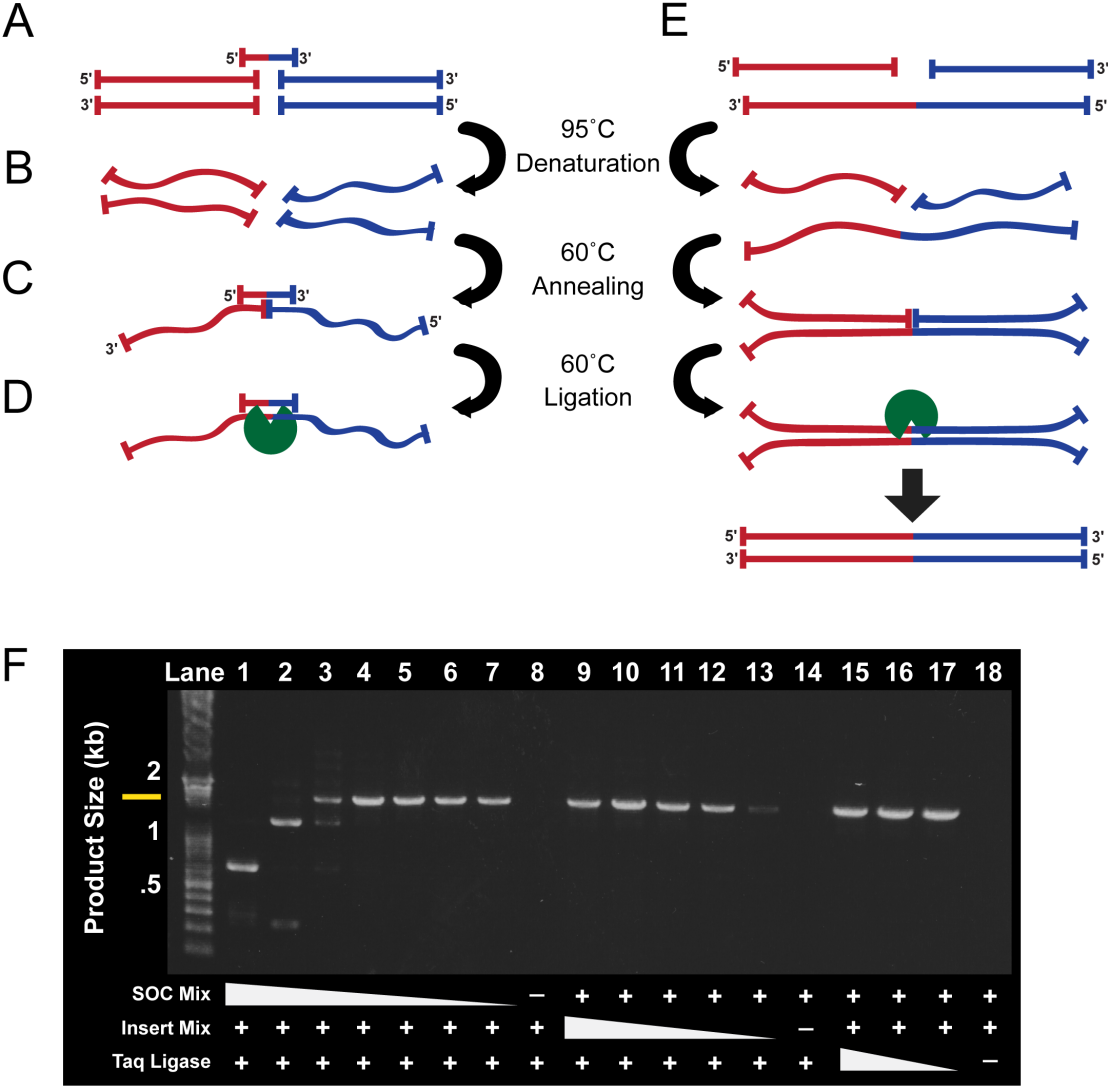
Cycled Ligation Assembly Reaction. (**A**) Diagram of the assembly of two double-stranded DNA fragments (red and blue) using a Scaffold Oligonucleotide Connector (SOC). (**B**) DNA fragments are denatured at 95°C in a thermocycler instrument. (**C**) The reaction is cycled to 60°C, allowing a SOC homologous to the last 20 bp of the first DNA fragment and the first 20 bp of the second fragment to anneal, forming a transient partly double-stranded (ds) DNA structure. (**D**) A thermostable ligase (green) specific for nicks in dsDNA seamlessly ligates the two bottom strands. (**E**) Subsequent cycles of denaturation and annealing/ligation allow the thermostable ligase to ligate the top strands, using the ligated bottom strands as a template. Further cycles of ligation exponentially increase the amount of correct product as the products of early rounds served as an expanding pool of template for later cycles. (**F**) Successful assembly of three ∼0.6 kb DNA inserts (**Table 1**) using a cycled ligation assembly was confirmed by PCR amplification of a correctly sized 1.8 kb fragment (yellow bar). A range of concentrations of reaction components was tested in order to find optimal conditions. SOC concentration (ten-fold serial dilutions from 10 µM to 10 pM, lanes 1–7), inserts concentration (6 nM, 2 nM, 0.6 nM, 0.2 nM, 0.06 nM, lanes 9–13), and Taq Ligase concentration (240 U, 80 U, 24 U, lanes 15–17) were varied (+ denotes optimal concentrations of 10 nM SOCs, 2 nM inserts, and 80 U taq Ligase).

A Cycled Ligation Assembly (CLA) reaction requires 5′ phosphorylated insert fragments of DNA, a thermostable ligase, a buffer, and short oligonucleotides termed Scaffold Oligonucleotide Connectors, or SOCs. A SOC is a 40 bp synthetic oligonucleotide homologous to the final 20 bp sequence of the first DNA fragment to be assembled and the first 20 bp of the second fragment (**Figure 1A**). One SOC is designed for each joint.

The design of a cycled ligation assembly (Figure 1) takes advantage of a thermostable ligase specific for nicks in double-stranded DNA (e.g. Taq Ligase [19]). The thermostability enables multiple rounds of denaturation and ligation. Two DNA fragments, for example encoding a promoter and gene, or a protein of interest and a fluorescent protein, are PCR-amplified with 5′ phosphorylated primers. The 5′ phosphorylation is required for Taq Ligase to form a new phosphodiester bond between two inserts. The two fragments (Red and Blue, **Figure 1**) are ligated directly together in a single reaction without the need for restriction digests, vectors, or any modifications to their nucleotide sequences. First, the two fragments are denatured (**Figure 1B**) in the presence of a suitable SOC, which allows the SOC access to the complementary strands of the two sequences when the reaction is returned to an annealing temperature (**Figure 1C**). Binding of the SOC to the complementary strands of the two fragments connects them for assembly, creating a temporary dsDNA structure with a single phosphodiester bond missing between the two complementary strands (**Figure 1C**). The thermostable Taq Ligase specifically ligates the complementary strands together to produce a continuous ssDNA structure with no lost or added nucleotides at the joint (**Figure 1D**). SOCs serve only as initial scaffolds for fragment assembly, with successfully assembled products becoming additional templates as they are created (**Figure 1E**). The two ligated strands anneal to their complementary strands, again creating a single nick in a dsDNA structure (**Figure 1E**). The amount of correctly assembled product increased exponentially as the reaction cycles between denaturing and annealing/ligation temperatures.

We optimized and standardized a single set of concentrations for inserts, SOCs, and Taq ligase (**Figure 1F**) to obtain maximally efficient assembly of the correct product. This standardized assembly protocol was used for all further experiments. Omitting either SOCs, ligase, or buffer prevented any detectable assembly (**Figure 1F** **, lanes 8, 14, 18**), as did the absence of 5′ phosphates from DNA insert sequences.

The molar concentration ratio of SOCs to insert strands proved critical. Too few SOCs in the reaction made early assembly events unlikely, while too many SOCs interfered with assembly in later cycles. With a 5∶1 molar ratio, optimal SOCs are needed only to initiate the assembly reaction. Once opposite strands are ligated, they serve as templates for assembling additional complementary strands. In early cycles SOCs were required to initiate the assembly of the complementary strands (**Figure 1A–D**). In later cycles (**Figure 1E**), if SOCs are bound to already-ligated strands they compete with productive assembly of complementary strands. The 5∶1 ratio (**Figure 1F** **, lanes 4**) is the optimal balance between these conflicting requirements.

### Cycled Ligation Assembly of Many Inserts

**Figure 2:**
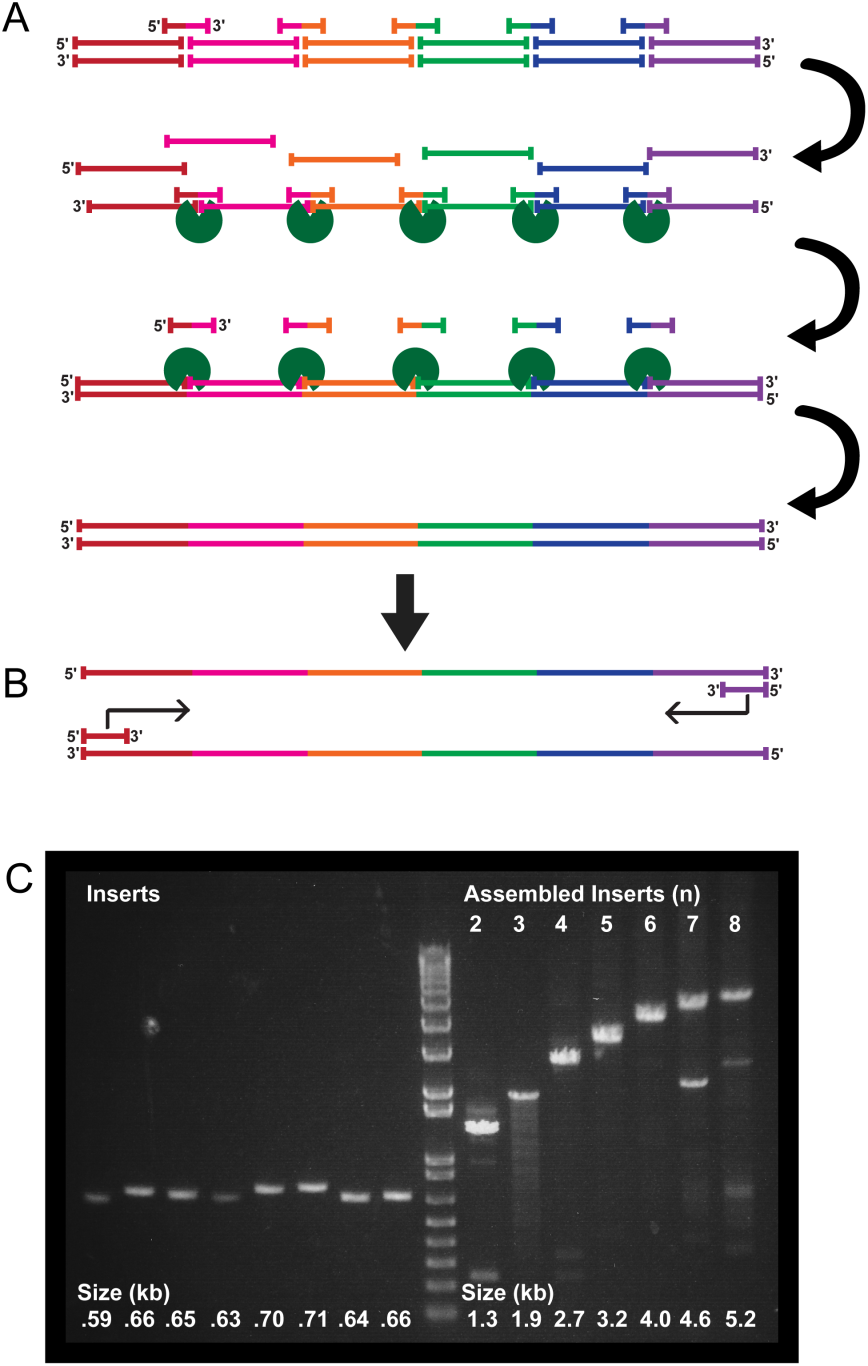
Directed Assembly of Many DNA Fragments in a Single Reaction. (**A**) A large number of DNA fragments can be assembled in a single reaction. The order of the inserts is determined solely by the included SOCs. Denaturation and renaturation allows SOCs to link fragments that become substrates for ligase (green). Further cycles of denaturation and renaturation, without any polymerase activity, assemble the desired double-stranded construct. (**B**) PCR amplification of the assembly product using a forward primer for the first fragment and a reverse primer for the last fragment yields large amounts of correctly assembled product. (**C**) Gel electrophoresis of PCR amplification of cycled ligation assembly products. Eight ∼650 bp DNA sequences (**Table 1**) were successfully assembled in a single reaction, in a pre-determined order and orientation, and subsequently amplified. The number of inserts (n) assembled into each product is displayed above lanes. The size (in kb) of assembled products is displayed below each lane. Products greater than 5 kb were successfully assembled in a defined order from eight DNA fragments.

Many more than three DNA fragments can be ligated in a specific order and orientation using a single CLA (**Figure 2A**). The success in such reactions is due to the specificity of Taq Ligase for nicks in dsDNA, the specificity of each SOC for only two DNA fragments aligned in a certain order, and the exponential increases in template and product from cycling. We tested multi-fragment assembly using eight fragments of DNA amplified from protein-coding regions (**Table 1**), each approximately 650 bp in length. SOCs were designed to specify the order in which the DNA fragments were ligated (**Figure 2A**). Ligated single-stranded sequences then directed selective ligations of complementary strands. The final product was a seamlessly assembled array of the input fragments, with the order, orientation, and nucleotide sequence as designed (**Figure 2A, C**). The product can readily be amplified by further PCR as desired (**Figure 2B**). CLAs were successful in assembling as many as eight ∼650 bp DNA sequences in a user-determined order in a single reaction (**Figure 2C** **,** **Table 1**), demonstrating the complexity of assemblies achievable using the cycled-ligation approach. We sequenced the product and determined that it was perfect and scarless.

**Table 1:** DNA Fragments Used in Cycled Ligation Assembly.

Gene	Coordinates	Size (bp)	Figure
*SNAP*	NA	594	1, 2
*SuFu*	NM_001025391 pos 543–1202	660	1, 2
*Smoothened*	NM_176996 pos 1911–2560	650	1, 2
*TMEM 231*	NM_001033321 pos 26–652	627	2
*NPC1*	NM_008720 pos 2013–2708	696	2
*GFP*	NA	714	2
*Gli2*	NM_001081125 pos 1468–1940,2063–2105	515	2
*Patched*	NM_008957 pos 1443–2106	664	2
*Patched*	NM_008957 pos 1–321	321	3A
*Patched*	NM_008957 pos 322–1266	945	3A
*Patched*	NM_008957 pos 1267–1752	486	3A
*Patched*	NM_008957 pos 1753–2202	450	3A
*NPC1*	NM_008720 pos 3441–3518	78	3A
*Patched/YFP*	NM_008957 pos 3040–4302	2061	3A
*SNAP*	NA	122	3B, 3C, 4
*SuFu*	NM_001025391 pos 208–357	150	3B, 3C, 4
*Gli2*	NM_001081125 pos 90–223	134	3B, 3C, 4
*Inversin*	NM_010569 pos 245–363	119	3B, 3C, 4
*TMEM 231*	NM_001033321 pos 20–183	164	3B, 3C, 4
*Smoothened*	NM_176996 pos 670–831	162	3B, 3C, 4
*Dendra2*	NA	131	3B, 3C, 4
*SNAP*	NA	106	3B, 3C, 4
*SuFu*	NM_001025391 pos 1522–1662	141	3B, 3C, 4
*Gli2*	NM_001081125 pos 4865–4981	135	3B, 3C, 4
*Inversin*	NM_010569 pos 3304–3425	132	3B, 3C, 4
*TMEM 231*	NM_001033321 pos 849–971	125	3B, 3C, 4
*Smoothened*	NM_176996 pos 2767–2891	148	3B, 3C
*Dendra2*	NA	141	3B, 3C
Pericentrin	XM_005261124 pos 9434–9531	143	3B, 3C
GFP	NA	139	3B, 3C

**Figure 3:**
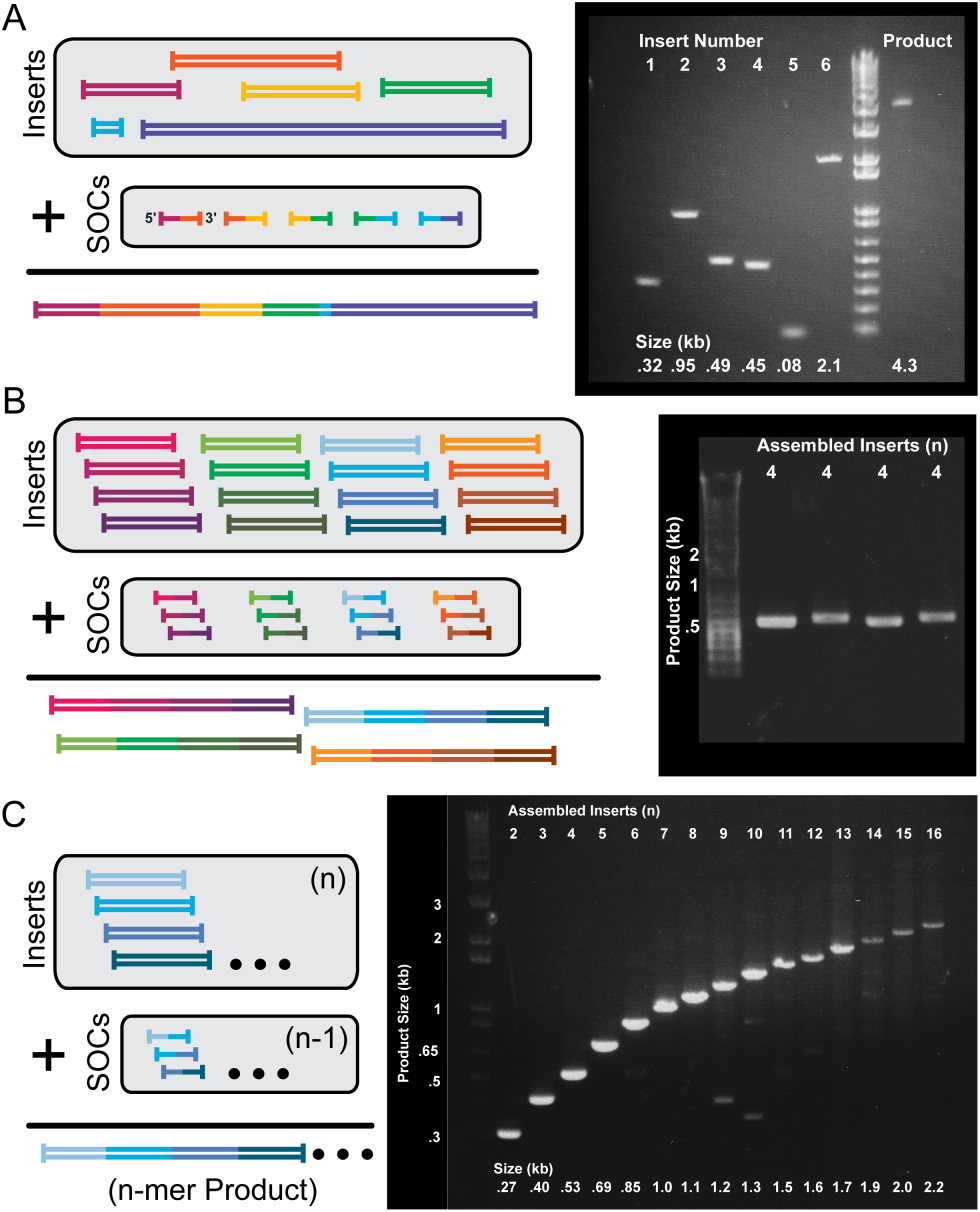
Cycled Ligation Enables Both Complex and Multiplexed Assemblies. (**A**) Complex assembly of very small (80 bp) and large (2100 bp) DNA fragments (**Table 1**) into a single product. Six DNA fragments of various sizes were seamlessly assembled in a single reaction and amplified by PCR. (**B**) Sixteen 100–200 bp DNA sequences (**Table 1**) were placed in a single cycled ligation reaction. Twelve SOCs were added to guide the assembly of four different products consisting of four inserts each (three SOCs per product). Each of the four products was PCR amplified from the single cycled ligation reaction, demonstrating that multiplexed assembly reactions work well. (**C**) Assembly of sixteen distinct DNA fragments into a single ordered product in a single reaction. The number of inserts (n) assembled into each product is displayed above lanes. All sequences, primers, and SOCs described in **Tables S1** and **S2**.

**Figure 4:**
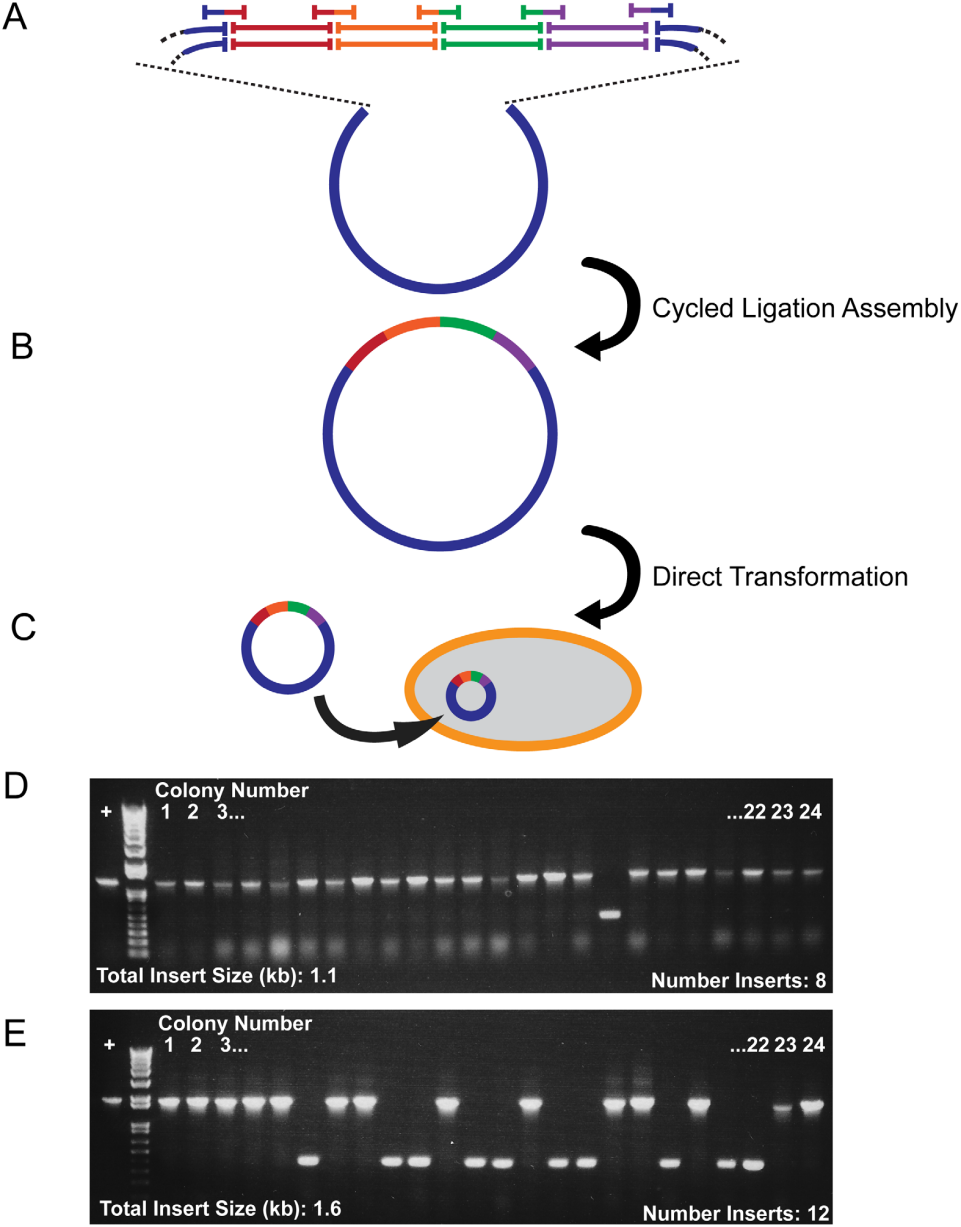
Single-step Assembly of Many Inserts Into a Transformable Plasmid. (**A**) Schematic of SOC-mediated cycled-ligation assembly of multiple inserts directly and seamlessly into a blunt-digested vector. The vector was digested with two different blunt restriction enzymes to minimize background colonies from uncut vector. Addition of SOCs bridging the blunt vector ends and first and last inserts directed the assembly of a circular cycled-ligation assembly product. (**B**) Seamlessly assembled circular plasmid. (**C**) Plasmid products of cycled-ligation assembly were directly transformed into competent cells for amplification. Assembly reactions were pipetted into a competent cell mixture. (**D, E**) Successful colony PCR resulted from assembly of eight (**D**) or twelve (**E**) 100–200 bp inserts (**Table 1**) into a double blunt-digested 3.1 kb cloning vector (**Table S1**). Over 95% (**D**) and 50% (**E**) of colonies were positive for the correct sizes of the eight or twelve inserts. Colonies 1 through 3 were grown and their DNA sequenced. All three sequenced colonies had the correct order of inserts and no mutations. Results are representative of at least three independent experiments.

DNA fragments amplified from specific protein-encoding domains used in Figures 1 and 2, Figure 3A, and Figures 3B, 3C, and 4.

### Applications of Cycled Ligation Assembly

Without changing or optimizing reaction conditions for each new assembly reaction, we applied cycled ligation assemblies to successfully assemble DNA fragments of a wide range of sizes. CLAs could assemble very small (<100 bp) and large (>2 kb) DNA sequences in a single reaction (**Figure 3A** **,** **Table 1**), a critical advantage compared to Gibson Assembly, which cannot assemble small DNA sequences due to the presence of an exonuclease in the reaction. To more fully test the specificity of the CLR we prepared a single assembly reaction containing sixteen 100–200 bp DNA fragments. The fragments contained sequences from N- and C-Terminal Domains from nine proteins (**Table 1**). By adding different sets of three SOCs, subsets of the 16 fragments were assembled into four separate products, each containing four of the original 16 fragments. Four PCR reactions were used to amplify the final products from the single CLA reaction; by using different primers only one product was amplified in each reaction (**Figure 3B**). Their exact structures were confirmed by cloning and sequencing. This demonstrates the potential to multiplex a DNA assembly reaction, with the specificity of SOCs ensuring that multiple separate products can be assembled in the same reaction without interfering with each other. CLAs were even successful in assembling sixteen ∼150 bp DNA sequences in a user-determined order in a single reaction (**Figure 3C**). Each lane shows the products of a reaction containing increasing numbers of relevant SOCs, with just one SOC used to make the products in lane 2 and 15 SOCs used for the products in lane 16. The results show that few reactions are partial, since each lane contains one prominent band, and that progressive addition of more SOCs did not interfere with the reactions of the other SOCs. These results demonstrate the utility of using the cycled-ligation approach to the assembly of complex constructs.

### Direct Assembly of Transformable Plasmids

A CLA further increases the utility and efficiency of complex genetic recombination experiments when used to directly introduce single or multiple fragments into a transformable vector. By including SOCs joining the first and last inserts to blunt cut sites on a vector (**Figure 4A**), multiple DNA sequences can be assembled and inserted into a vector, with the order and orientation of inserts determined by the SOCs used. The final product contains seamlessly integrated inserts (**Figure 4B**), and the reaction mixture can be directly pipetted into competent bacterial cells for transformation (**Figure 4C**). To demonstrate the complexity of DNA assemblies enabled in a CLA, we assembled eight ∼150 bp DNA sequences into a blunt-digested 2.7 kb cloning vector and directly transformed chemically competent cells with the reaction product (**Table 1**). A vector digested with two blunt restriction enzymes was used to reduce the recovery of plasmids without inserts that arise from uncut vector. Restriction enzymes that created sticky ends were not used as Taq ligase readily re-ligated linearized sticky end digested vectors, although they could be used at a higher background with an additional 5′ dephosphorylation step. Colony PCR analysis of DNA from 24 randomly chosen colonies, out of hundreds of colonies obtained, revealed that >90% of colonies were positive for all eight inserts (**Figure 4D**). Three positive colonies per transformation were sequenced; no mutations or incorrect insertions were detected (data not shown). Twelve ∼150 bp fragments could be inserted directly into a transformable vector with greater than 50% of colonies positive for the full-length assembled insert (**Figure 4E** **,** **Table 1**).

## Discussion

The ideal DNA assembly technique for a given cloning project depends on a number of factors such as the complexity of the desired construct, sizes of fragments to be assembled, rates of throughput needed, experience with molecular biology, and cost constraints. Entire custom genes can be generated by synthesis [4], but the cost and/or skill required presently prevents widespread and frequent use of that approach. Most genetic constructs constructed in biomedical research labs are made up of protein-coding and regulatory DNA fragments that can be readily PCR-amplified from existing clones. Traditionally, the fragments can be recombined, one insert at a time, into various vectors using restriction digestions. Recent in vitro assembly methods, Golden Gate Assembly [5] and Gibson Assembly [8], improve upon the capabilities of restriction digest-based assemblies, but each has drawbacks as well.

We present a cycled-ligation assembly reaction that offers unique advantages over other current genetic assembly techniques. Restriction digests, Golden Gate Assembly, Gibson Assembly, and additional methods such as Overlap Extension-PCR [20], all require exogenous sequences to be added to inserts before assembly. In contrast, the sequences that are PCR-amplified for a CLA do not require any nucleotide additions. The cumbersome and error-prone need to introduce restriction sites or homologous regions into inserts is eliminated. Instead the specific order of inserts in the assembly is encoded in the DNA sequences of small, synthetic, oligonucleotides termed Scaffold Oligonucleotide Connectors, or SOCs.

Easily designed Scaffold Oligonucleotide Connectors (containing the last 20 bp of the first sequence and the first 20 bp of the next) guide and direct the assembly, but are not incorporated into the final product. The same amplified starting fragment can be reused from one DNA assembly plan to the next, with only the SOCs changing to guide alternative constructions. A given sequence (such as for GFP) need only be amplified once to be assembled into different locations in any number of constructs. This is in stark contrast to comparable assembly strategies, where each insert must encode its own assembly instructions through attached restriction sites or homologous sequences, hampering the reusability of a sequence from one assembly reaction to the next.

CLAs reduce the potentially error-prone addition of nucleotides by a DNA polymerase during the assembly reaction, a feature in common with restriction site-based assembly but distinct from Gibson assembly. During the assembly reaction, the input insert sequences are simply ligated together in an order specified by the included SOCs, reducing the probability that mutations will be introduced during the splicing. Some mutations may be introduced during PCR amplification of insert sequences, as with all procedures that use PCR.

The specificity of CLAs even allows multiple distinct assembly reactions to take place simultaneously in the same tube. Inclusion of a vector backbone in a CLA enables the direct transformation of assembled circular constructs into competent cells immediately following assembly. However, CLAs are not ideal in all situations. Due to cycles of denaturation and annealing, highly homologous insert sequences can interfere with each other’s assembly, reducing efficiency. Gibson assemblies share this limitation, whereas Golden Gate Assemblies are especially well suited for working with homologous sequences. When using CLAs to directly assemble inserts into a plasmid, the plasmid must be linearized using blunt ended restriction digestions due to non-productive re-ligation of sticky ends by Taq ligase, a limitation not found in Gibson Assemblies due to its lower Taq ligase concentrations [8]. Golden Gate Assemblies are better suited for high throughput applications than CLAs or Gibson assemblies.

The efficiency of CLAs decreases as the size of the assembled DNA fragments grows above 5–6 kb. This effect is likely due to the probabilistic nature of CLAs, where productive assembly is due to four-part interactions between two single stranded DNA fragments, a SOC, and Taq ligase. As the length of DNA fragments increases, these rare events are crowded out by the higher probability of off-target binding between various combinations of SOCs and DNA fragments. However, other established methods, such as Gibson assemblies or Successive Hybridization Assemblies (SHA), excel in the assembly of these larger fragments [8], [21]. CLAs can be combined with such methods for hierarchical assembly strategies where many smaller DNA fragments are assembled using CLAs into medium sized products for use in Gibson Assemblies or SHAs.

At the scale of the most common DNA assembly goals, using fragments from <500 bp to 5 kb, cycled-ligation assemblies are efficient and powerful, able to assemble many inserts in a single reaction. Expanding on prior ligase-based cloning methods [18], we present a single, optimized CLA protocol which works across these scales, with set reaction parameters that need not be optimized from one reaction to another. We have demonstrated the utility of CLA by applying this single protocol to the assembly of numerous complex genetic constructs.

CLAs are simple to design and set up, take under two hours, and are cheaper per reaction than other methods. They give the experimentalist complete control over the final product sequence. CLAs should enable any lab that does PCR reactions to embark on, and accomplish, intricate multi-insert assemblies at medium throughput with great efficiency. Such constructs provide invaluable tools for genetic manipulations required to answer many basic science and biomedical questions.

## Materials and Methods

### Cycled-ligation Assembly Protocol

All cycled-ligation assembly reactions described were carried out using the following standardized protocol (**Figure 1**). The sequences of the vectors and inserts used in this study are presented in **Table S1**. All scaffold oligonucleotide connector (SOC) and primer sequences are presented in **Table S2**. Mixtures, incubations, and thermocycler programs are presented in **Table S3**.

### 1) Scaffold Oligonucleotide Connector Design

SOCs were designed by combining the final 20 bp of one DNA insert or vector and the first 20 bp of the adjacent insert or vector in the desired assembled product. The resulting 40 bp oligonucleotides were synthesized for us by Elim Biopharmaceuticals.

### Primer Phosphorylation

DNA inserts for cycled ligation reactions were amplified by PCR. Primers for PCR reactions were 5′ phosphorylated with T4 Polynucleotide Kinase (NEB). T4 Ligase Buffer is used to supply ATP. Detailed protocol in **Table S3**.

### 2) DNA Insert PCR Amplification

The primer phosphorylation mixture was directly added to a PCR reaction to amplify insert sequences containing 5′ phosphorylated ends. A high fidelity polymerase (Phusion, NEB) was used to reduce the introduction of mutations. PCR products were analyzed by gel electrophoresis (1% agarose gel) and correctly sized bands were isolated by gel extraction (Qiagen). Detailed protocol in **Table S3**.

### 3) Double Blunt Vector Digestion

For direct assembly of transformable plasmids by cycled ligation, a modified pUC19 vector (**Table S1**) with two blunt ligation sites separated by ∼250 bp was created. The vector was digested with NruI and SwaI (NEB) and the linearized vector was isolated and purified by gel extraction (Qiagen). Detailed protocol in **Table S3**.

### 4) Cycled Ligation Reaction

Linearized vector was diluted in H_2_O to a concentration of 5 nM. The desired inserts for a specific cycled ligation assembly reaction were mixed and diluted in H_2_O to form an insert mixture with a concentration of 20 nM for each insert. 1 µl of each scaffold oligonucleotide connector (200 µM) used in a specific cycled-ligation assembly reaction was added into 100 µl of H_2_O, and this SOC mixture was further diluted 1∶10 in H_2_O (200 nM) before use. For Cycled Ligation Assemblies without a vector, the volume of the linearized vector was replaced with H_2_O. Mixture volumes and thermocycler program are presented in **Table S3**.

#### 5-1) PCR Amplification of Assembled Products

The amount of assembled product from a cycled ligation assembly is in the nM-pM range, and usually must be amplified for subsequent applications if it is not directly assembled into a vector. The products of cycled ligation reactions were PCR-amplified with a high fidelity polymerase (Phusion, NEB). After assembly, the cycled ligation reaction mixture was directly added to a PCR reaction to amplify the desired assembled product. In some cases multiple different products were amplified from the same cycled ligation reaction (**Figure 3B**). Amplification products were analyzed by gel electrophoresis. Amplification at this stage can be used to add restriction sites or homologous regions if the assembled product is to be inserted into a vector using a restriction digestion or Gibson Assembly. Detailed protocol in **Table S3**.

#### 5-2) Transformation of Assembled Plasmid Products

Plasmid products of cycled ligation reactions were directly transformed into chemically competent bacteria (One Shot TOP10, Invitrogen) according to the manufacturer’s protocol. Briefly, 2 µl of the cycled ligation reaction mixture was added to 50 µl of competent cells on ice. After 30 min on ice, the cells were placed in a 42°C water bath for 30 sec and then on ice for 2 min. 250 µl of pre-warmed (37°C) SOC medium (Invitrogen) was added and the cells were incubated for 1 hr at 37°C and 225 RPM. 250 µl were spread onto a pre-warmed LB+Ampicillin 10 cm plate. Plates were incubated at 37°C overnight.

### Colony PCR and DNA Sequencing

To screen for positively assembled products, colonies from plates incubated overnight were randomly picked for colony PCR. After manually counting the number of colonies per plate, each colony was picked with a 200 µl pipette tip and directly added into the PCR mix described below. Using the same tip, the colony was inoculated into 500 µl LB medium for further growth. Primers (**Table S2**) corresponding to sequences ∼50 bp upstream of the first insert and 50 bp downstream from the last insert were used to amplify the size of the inserted sequence. Colony PCR results were analyzed by gel electrophoresis, and at least three positive colonies per plate were further grown overnight in 5 mL of LB medium + Ampicillin (100 µg/mL). Plasmid DNA was isolated by minipreps (Qiagen) according the manufacturer’s protocol, and the entire length of the insert region was sequenced (Elim Biopharmaceuticals). Detailed protocol in **Table S3**.

## Supporting Information

Table S1 **Sequences of DNA Fragments and Vectors.** This table contains the sequences of the DNA fragments used in Figures 1–4 and described in **Table 1**, and the sequence of vector used in Figure 4.
(XLSX)

Table S2 **Sequences of Primers and SOCs.** This table contains the sequences of primers and SOCs used in Figures 1–4.
(XLSX)

Table S3 **Cycled Ligation Assembly Mixtures, Incubations, and Thermocycler Programs.** This table contains a detailed protocol for each step of the cycled ligation assembly reaction.
(XLSX)
